# Modulation of intestinal microbiota, morphology and mucin composition by dietary insect meal inclusion in free-range chickens

**DOI:** 10.1186/s12917-018-1690-y

**Published:** 2018-12-04

**Authors:** Ilaria Biasato, Ilario Ferrocino, Elena Biasibetti, Elena Grego, Sihem Dabbou, Alessandra Sereno, Francesco Gai, Laura Gasco, Achille Schiavone, Luca Cocolin, Maria Teresa Capucchio

**Affiliations:** 1 Department of Veterinary Sciences, University of Turin, Largo Paolo Braccini 2, 10095 Grugliasco, TO Italy; 2 Department of Agricultural, Forest and Food Sciences, University of Turin, Largo Paolo Braccini 2, 10095 Grugliasco, TO Italy; 3 Institute of Science of Food Production, National Research Council, Largo Paolo Braccini 2, 10095 Grugliasco, TO Italy; 4 Institute of Multidisciplinary Research on Sustainability, University of Turin, Via Accademia Albertina 13, 10100 Turin, Italy

**Keywords:** 16S rRNA, Gut health, Microbiota, Morphometry, Mucin, Poultry, *Tenebrio molitor*

## Abstract

**Background:**

Gut health in poultry depends on the balance between the host, intestinal microbiota, intestinal microscopic features and diet. The effects of insect meal (a promising alternative protein source for poultry feed) on chicken gut morphology have recently been reported, but no data about intestinal microbiota and mucin composition modulation are available. The present study evaluated the effects of dietary *Tenebrio molitor* (TM) meal inclusion on gut health of free-range chickens by intestinal microbiota, morphology and mucin composition characterization.

**Results:**

One hundred forty female medium-growing hybrids were divided into 2 dietary treatments (control feed [C] and 7.5% TM inclusion, with 5 replicate pens/treatment and 14 birds/pen) and slaughtered at 97 days of age (2 birds/pen for a total of 10 chickens/diet). The gut microbiota assessment on cecal content samples by 16S rRNA amplicon based sequencing showed higher alpha (Shannon, *P* < 0.05) and beta (Adonis and ANOSIM, *P* < 0.001) diversity in birds fed TM diet than C. In comparison with C group, TM birds displayed significant increase and decrease, respectively, of the relative abundances of *Firmicutes* and *Bacteroidetes* phyla, with higher *Firmicutes:Bacteroidetes* ratios (False Discovery Rate [FDR] < 0.05). The relative abundance of *Clostridium*, *Oscillospira*, *Ruminococcus, Coprococcus* and *Sutterella* genera was higher in TM chickens than C (FDR *<* 0.05). On the contrary, TM birds displayed significant decrease of the relative abundance of *Bacteroides* genus compared to the C group (FDR < 0.05). Gut morphology evaluation by morphometric analysis on small intestine revealed similar villus height, crypt depth and villus height to crypt depth ratio between C and TM birds. Characterization of gut mucin composition by periodic-acid Schiff, Alcian Blue pH 2.5 and high iron diamine staining on small and large intestine showed unaffected mucin staining intensity in TM chickens when compared to C group.

**Conclusions:**

Dietary TM meal inclusion may positively modulate the gut microbiota of the free-range chickens without influencing the intestinal morphology and mucin composition. Since the rapid growth of chickens directly depends on morphological and functional integrity of the digestive tract, the gut health assessment by a post mortem multidisciplinary approach appears to be fundamental.

**Electronic supplementary material:**

The online version of this article (10.1186/s12917-018-1690-y) contains supplementary material, which is available to authorized users.

## Background

Gut health has been a focus of major research efforts in production animals, since it can be considered a synonymous to animal health and is of vital importance to animal performance [1]. The latter aspect has a key role in poultry industry, which requires animals capable of growing rapidly within a short period of time.

Gut health depends on the maintenance of the delicate balance between the host, intestinal microbiota, intestinal barrier (in terms of microscopic structure) and dietary compounds [2]. In particular, gut microbiota benefits the host by providing nutrients from otherwise poorly utilized dietary substrates and modulating the development and function of the digestive and immune system [3]. Firstly, gut microbiota can affect intestinal morphology through modifications of villus height and crypt depth [4], which are considered the main indicators of gut development, health and functionality [5]. There is also evidence that gut microbiota may modulate synthesis and composition of mucins [4], which constitute a digestion- and absorption-assisting medium and represent the first line of defense for intestinal epithelium against foreign bacteria and other pathogens [6]. Several feed substances have been reported to widely affect gut health in poultry, either by directly modifying intestinal morphology [7, 8] and mucin composition [9, 10], or indirectly by modulating intestinal microbiota [3, 11].

Feed may be considered the most important entity in the poultry industry in terms of animal health and producers revenue and profit. As a first aspect to consider, feed substances are capable of exposing the birds to potentially harmful organisms and/or components through the gastrointestinal tract [12]. Feed is also the major component of the total cost of production, with protein sources representing the primary one [13]. Corn and soybean meal constitute the main ingredients of choice for poultry diets worldwide [12], but the search for more sustainable, less food-competing and alternative protein feedstuffs is progressively increasing [13].

Insect meal utilization as alternative protein source for animal feeding has already been pointed out [14, 15], because of the excellent nutritive properties, the low competitiveness with human food and the reduction of the environmental contamination [15]. Compared to the conventional protein feedstuff (in particular soybean meal), insects contain higher or similar protein content, being also richer in essential amino acids [14, 15]. Furthermore, mass production of insects is currently promising in an ecological perspective, since it can lead to a significant reduction of the environmental impact in terms of energy cost, land area utilization and footprints. Indeed, insects can easily grow on different organic side streams, whose elimination has an economic and environmental cost. As a consequence, insects rearing may promote an advantageous reutilization of by-products, also transforming waste into high-protein feed that can replace increasingly more expensive compound feed ingredients [15]. Among insect species, meal obtained from larvae of *Tenebrio molitor* (TM) has recently been tested in diets for broiler [16–20] and free-range chickens [21] as protein source partially or totally replacing the only soybean meal [16, 18], soybean meal, corn gluten meal and soybean oil together [19, 20], or corn gluten meal alone [21]. In particular, TM larvae meal has been reported to be a suitable dietary ingredient in terms of unaffected or partially improved growth performance, haematochemical parameters, carcass traits and histological features [16, 18–21]. The implications of TM larvae meal utilization have also been investigated on gut health by intestinal morphology assessment [19–21], but no data about the modulation of gut microbiota and mucin composition by their inclusion in poultry diets are currently available.

The present study aims to investigate the effects of TM meal utilization on intestinal microbiota, morphology and mucin composition of free-range chickens, also proposing a standardized multidisciplinary post mortem approach for the assessment of gut health in poultry when dietary modifications occur.

## Results

### Cecal microbiota characterization

A total of 530,550 raw reads (2x250bp) were obtained after sequencing. After joint and quality filtering, a total of 104,081 reads passed the filters applied through QIIME, with an average value of 10,408 reads/sample. In order to avoid biases due to different sequencing depths, all samples were rarefied at 3600 reads after raw read quality filtering.

**Fig. 1:**
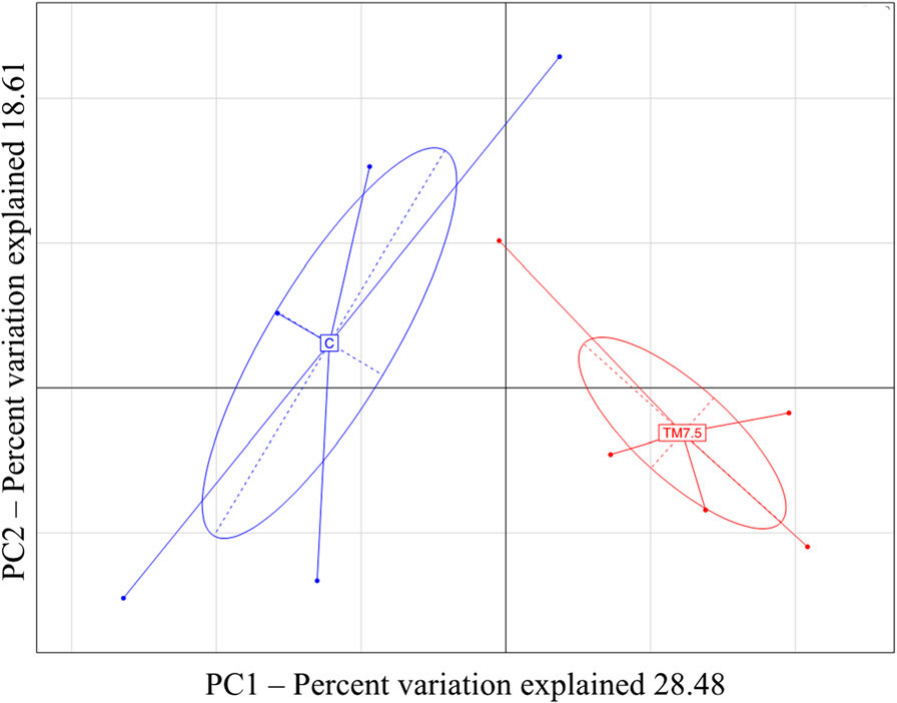
Bacterial community composition (weighted UniFrac beta diversity, PCA plots) in cecal samples of free-range chickens fed with control (C) and 7.5% inclusion level of *Tenebrio molitor* meal (TM7.5) diets. PC1 and PC2 components account for 28.48 and 18.61%, respectively, of the total variation (47.09)

The rarefaction analysis and the Good’s coverage indicated a satisfactory coverage for all the samples (average Good’s coverage of 84%) (Additional file 1). The diversity of the cecal microbiota between C and TM diets was assessed initially through measures of α-diversity and β-diversity. The Chao1, Phylogenetic Diversity (PD) Whole Tree and Shannon indexes and observed species richness were used to assess α-diversity (Additional file 1). Shannon index showed higher diversity (*P <* 0.05) in free-range chickens fed with TM compared to C diet, whereas Chao1 and PD Whole Tree indexes and observed species richness showed no significant differences (*P* > 0.05) between C and TM groups (Additional file 1). Weighted UniFrac distances were utilized as a measure of β-diversity to assess the effect of TM diet on bacterial community composition. Adonis and ANOSIM statistical tests based on Weighted UniFrac distance matrix showed significant differences among C and TM groups (*P* < 0.001). These differences were demonstrated by Principal Component Analysis (PCA), which showed a clear separation of the microbiota as a function of the diet (Fig. 1).

**Fig. 2:**
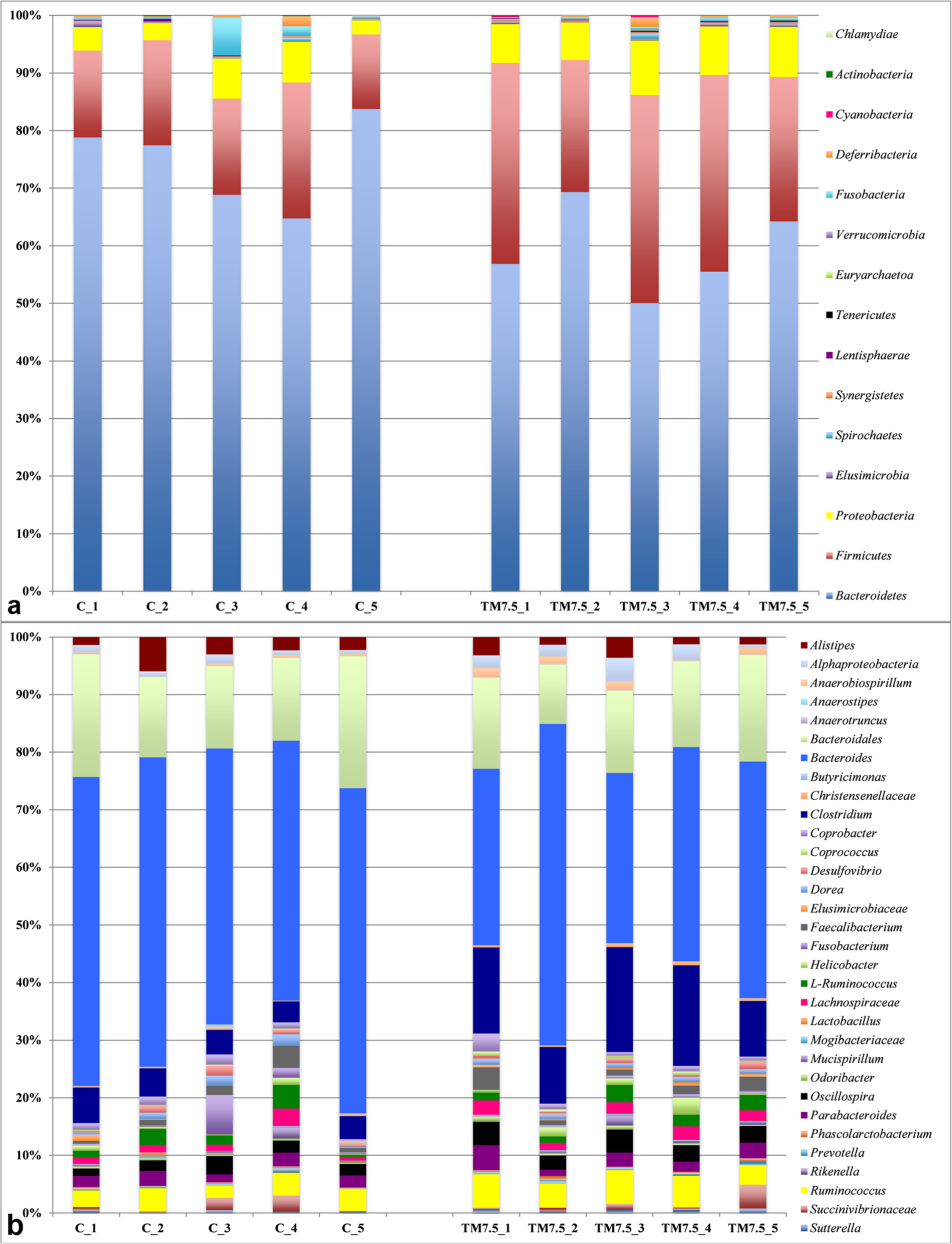
Relative abundance of the main bacterial phyla (**a**) and genera (**b**) in cecal samples of free-range chickens fed with control (C) and 7.5% inclusion level of *Tenebrio molitor* meal (TM7.5) diets. Taxa summary bar plots within the pooled cecal contents from the 5 replicate pens of control (C_1, C_2, C_3, C_4 and C_5) and 7.5% inclusion level of *Tenebrio molitor* meal (TM7.5_1, TM7.5_2, TM7.5_3, TM7.5_4 and TM7.5_5) dietary treatments

Relative abundances of the main phyla and genera in the free-range chickens of the present study obtained by 16S rRNA gene sequencing are summarized in Fig. 2 (phyla and genera per pools) and Additional file 2 (overall phyla and genera). *Bacteroidetes* represented the dominant phylum of the cecal community in both C and TM groups, outnumbering the *Firmicutes* and *Proteobacteria* phyla (Fig. 2a, Additional file 2). Within phylum *Bacteroidetes*, *Bacteroides*, unclassified members (U. m.) of *Bacteroidales* order, *Alistipes*, *Parabacteroides* and *Coprobacter* were identified as predominant OTUs in both birds fed with C and TM diets (Fig. 2b, Additional file 2). *Clostridium*, *Ruminococcus*, *Oscillospira*, L-*Ruminococcus*, *Faecalibacterium* and U. m. of *Lachnospiraceae* family were the dominant members of the *Firmicutes* phylum in both C and TM groups (Fig. 2b, Additional file 2). Within phylum *Proteobacteria*, U. m. of *Alphaproteobacteria* class and U. m. of *Succinivibrionaceae* family were observed as predominant OTUs in both chickens fed with C and TM diets (Fig. 2b, Additional file 2).

**Fig. 3:**
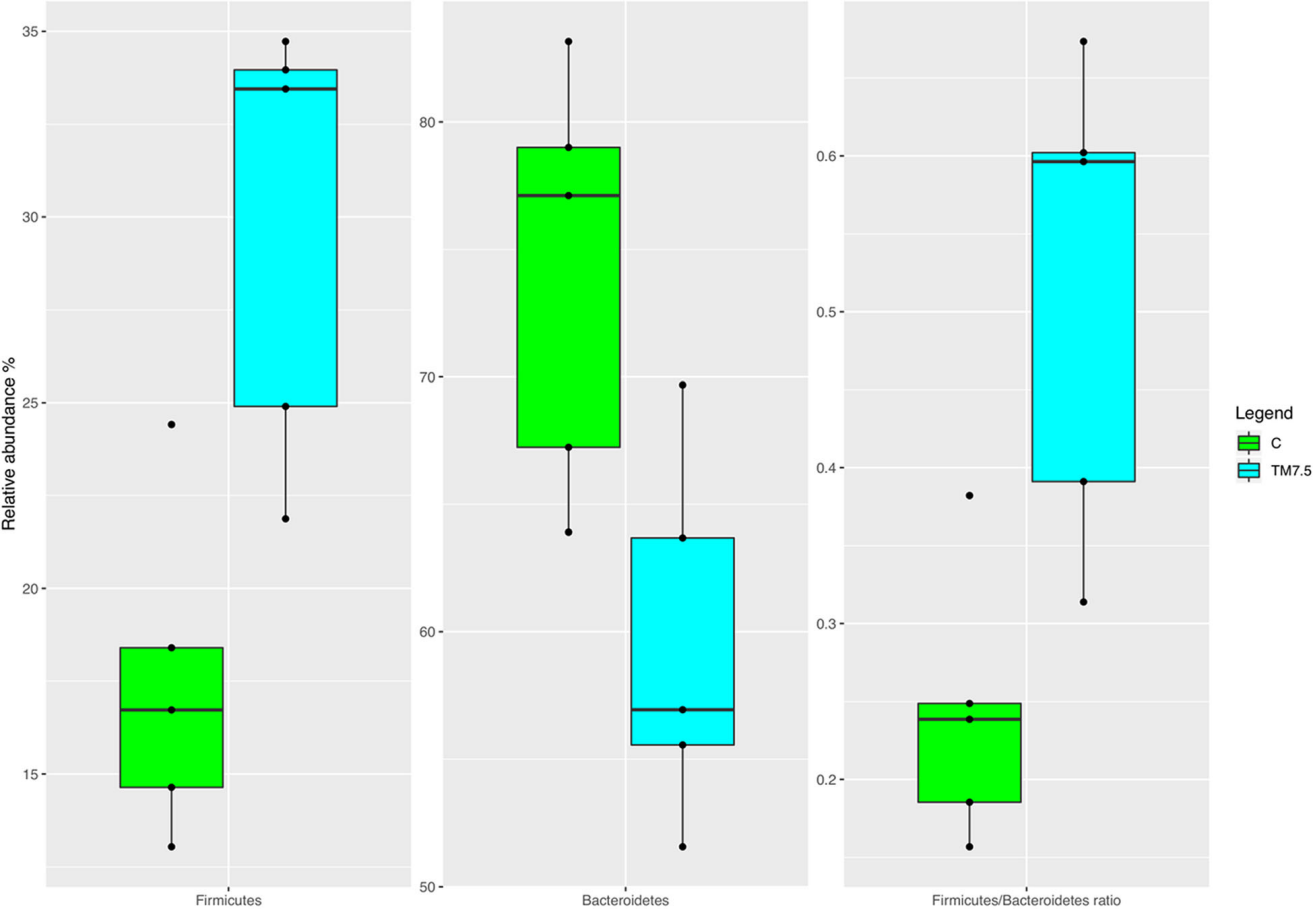
Boxplots showing the relative abundance at phylum level of OTUs differentially abundant based on Pairwise Kruskal-Wallis test (FDR < 0.05) in cecal samples of free-range chickens fed with control (C) and 7.5% inclusion level of *Tenebrio molitor* meal (TM7.5) diets

**Fig. 4:**
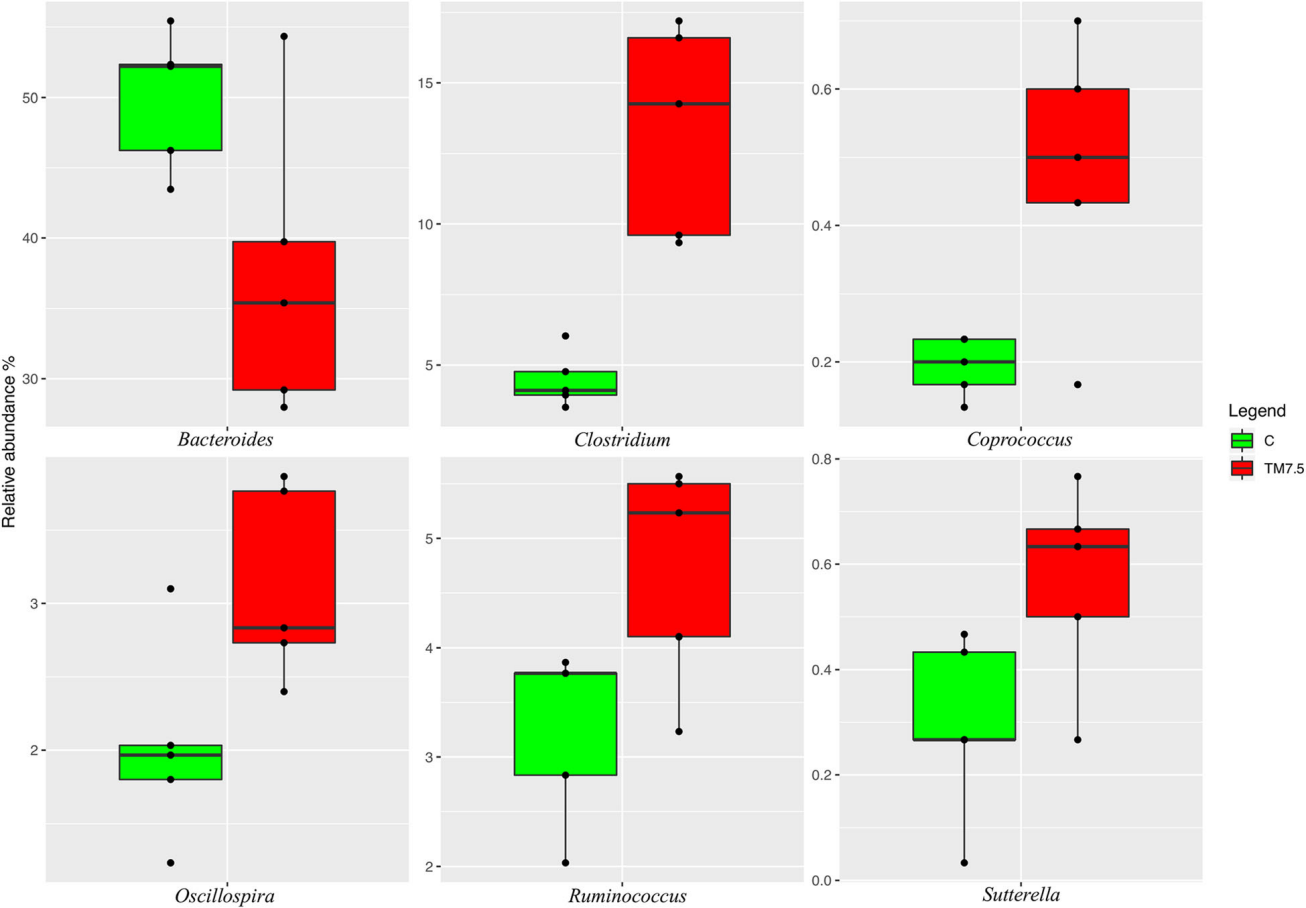
Boxplots showing the relative abundance at genus level of OTUs differentially abundant based on Pairwise Kruskal-Wallis test (FDR < 0.05) in cecal samples of free-range chickens fed with control (C) and 7.5% inclusion level of *Tenebrio molitor* meal (TM7.5) diets

Compared to the C group (Fig. 3), the birds fed with TM displayed significant increase of the relative abundance of *Firmicutes* phylum (FDR < 0.05). On the contrary, the relative abundance of *Bacteroidetes* was lower in TM chickens than C (FDR *<* 0.05). The birds fed with TM also showed higher *Firmicutes:Bacteroidetes* ratios compared to the C group (FDR < 0.05). At genus level (Fig. 4), the relative abundance of *Bacteroides* was lower in TM chickens than C (FDR < 0.05). On the contrary, the birds fed with TM displayed significant increase of the relative abundance of *Sutterella*, *Ruminococcus*, *Oscillospira* *, Clostridium* and *Coprococcus* genera compared to the C group (FDR < 0.05).

### Intestinal morphology

**Fig. 5:**
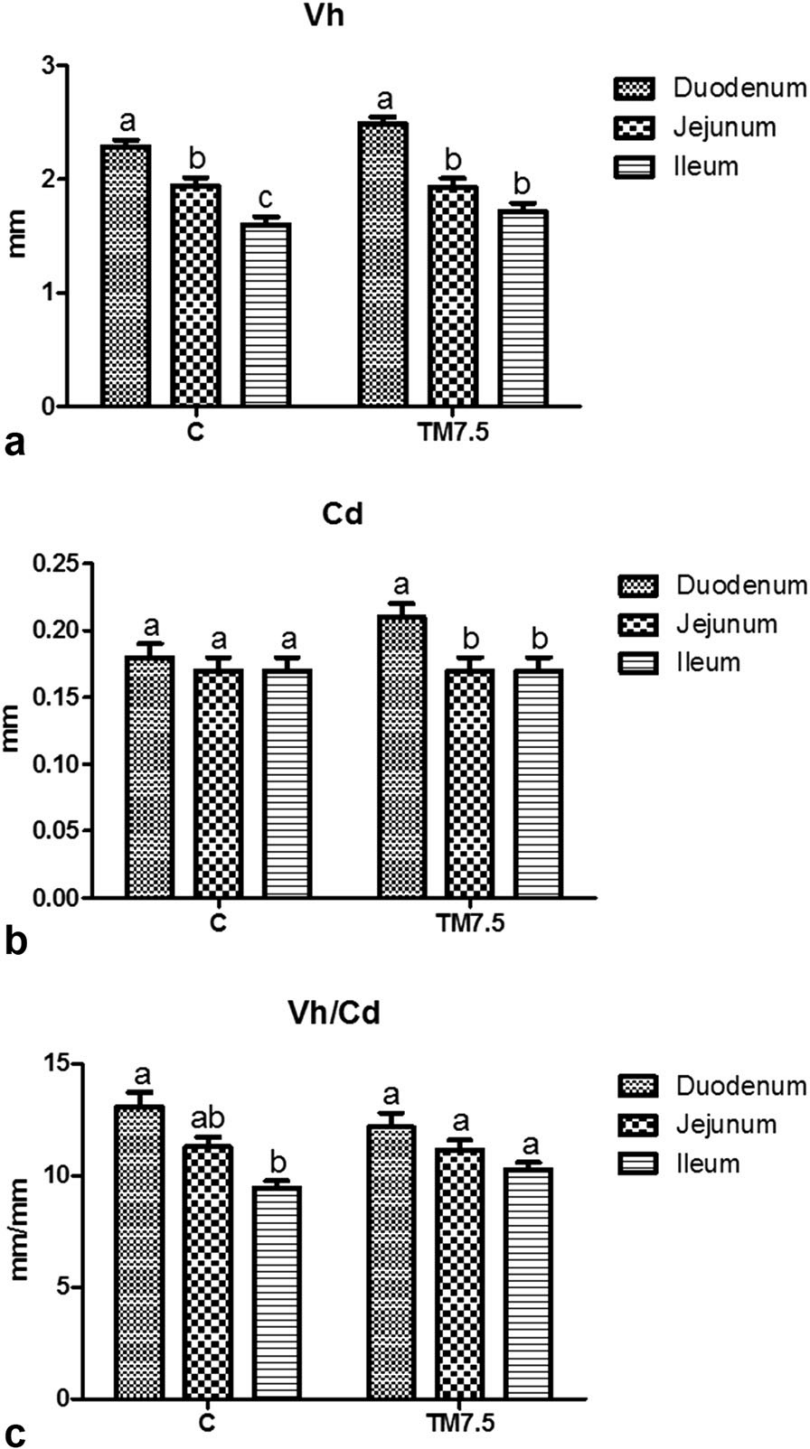
Graph bars of (**a**) villus height (Vh), (**b**) crypt depth (Cd) and (**c**) villus height to crypt depth ratio (Vh/Cd) in duodenum, jejunum and ileum of free-range chickens fed with control (C) and 7.5% inclusion level of *Tenebrio molitor* meal (TM7.5) diets. Graph bars with different superscript letters (a, b, c) within each dietary treatment differ significantly (*P* < 0.05)

Dietary TM meal inclusion did not affect the intestinal morphometric indices of the free-range chickens of the present study (*P* > 0.05, Fig. 5). Independently of TM meal utilization, the duodenum showed greater Vh (C and TM7.5 groups, *P* < 0.01) and Cd (TM7.5 group, *P* < 0.05) than the other gut segments and higher Vh/Cd ratio (C group, *P* = 0.01) than the ileum. The detailed results of the intestinal morphology of the chickens of the current trial are reported by Biasato et al. [21].

### Intestinal mucin composition

**Fig. 6:**
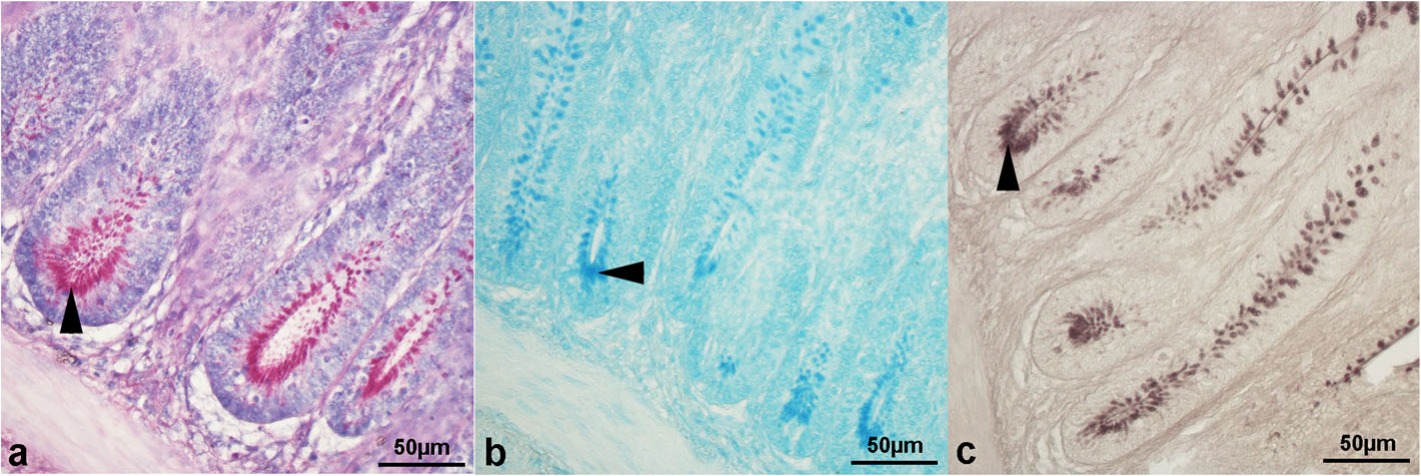
Histological pictures of (**a**) duodenal crypts stained with periodic-acid Schiff (40× magnification), (**b**) jejunal crypts stained with Alcian Blue pH 2.5 (40× magnification) and (**c**) ileal crypts stained with high iron diamine (40× magnification). Crypt bases (arrowheads) show higher mucin staining intensity than midsection and tip fragments

Mucin staining intensity in the intestinal crypts of free-range chickens of the present study significantly depended on mucin type, gut segment and crypt fragment (*P* < 0.001). However, there was no significant influence of dietary TM meal inclusion (*P* > 0.05) on the histochemical findings (Table 1). In particular, crypts showed higher acidic sialylated mucins staining intensity (*P* < 0.001) than neutral and acidic sulfated. Furthermore, lower mucin staining intensity was found in the caecal crypts (*P* < 0.001) compared with the other gut segments and in the jejunal crypts (*P* < 0.001) compared with the duodenum and ileum, respectively. Crypt base also showed greater mucin staining intensity (*P* < 0.001) than the midsection and tip (Table 2, Fig. 6).

**Table 1:** Effects of diet, mucin type, gut segment and crypt-villus fragment on mucin staining intensity in free-range chickens

Factor	d.f.^f^	Chi-square	*P*-value^g^
Crypts			
Diet^a^	1	0.77	ns
Mucin type^b^	2	34.61	^***^
Gut segment^c^	3	145.01	^***^
Fragment^d^	2	71.38	^***^
Villi			
Diet	1	0.00	ns
Mucin type	2	3.03	ns
Gut segment^e^	2	272.02	^***^
Fragment	2	10.99	^**^

^a^ Two dietary treatments: *C* control, *TM7.5* 7.5% inclusion level of *Tenebrio molitor*

^b^ Three types: neutral, acidic sialylated and acidic sulfated mucins

^c^ Four segments: duodenum, jejunum, ileum and cecum

^d^ Three fragments: base, midsection and tip

^e^ Three segments: duodenum, jejunum and ileum

^f^ Degrees of freedom

^g^ Statistical significance: *P* < 0.05 (^*^), *P* < 0.01 (^**^) and *P* < 0.001 (^***^). ns = not significant

**Table 2:** Least square means of mucin staining intensity in the intestinal crypts and villi of free-range chickens in relation to diet, mucin type, gut segment and fragment

	Factor	Factor levels	Mucin staining intensity^a,b^
Crypts	Diet	C	1.11 ± 0.03
		TM7.5	1.14 ± 0.03
	Mucin type	Neutral	1.04 ± 0.03^b^
		Acidic sialylated	1.28 ± 0.04^a^
		Acidic sulfated	1.06 ± 0.03^b^
	Gut segment	Duodenum	1.38 ± 0.04^a^
		Jejunum	1.05 ± 0.04^b^
		Ileum	1.31 ± 0.04^a^
		Cecum	0.83 ± 0.03^c^
	Fragment	Base	1.36 ± 0.04^a^
		Midsection	1.03 ± 0.03^b^
		Tip	1.01 ± 0.03^b^
Villi	Diet	C	1.65 ± 0.04
		TM7.5	1.65 ± 0.04
	Mucin type	Neutral	1.63 ± 0.05
		Acidic sialylated	1.72 ± 0.05
		Acidic sulfated	1.61 ± 0.05
	Gut segment	Duodenum	1.09 ± 0.04^c^
		Jejunum	1.91 ± 0.06^b^
		Ileum	2.16 ± 0.06^a^
	Fragment	Base	1.78 ± 0.05^a^
		Midsection	1.64 ± 0.05^ab^
		Tip	1.55 ± 0.05^b^

*C* control, *TM7.5* 7.5% inclusion level of *Tenebrio molitor*

^a^ Data are represented as mean of counts ± SEM

^b^ Means with different superscript letters (a, b, c) within the same column per factor (i.e. diet, mucin type, gut segment or fragment) differ significantly (*P* < 0.01)

**Fig. 7:**
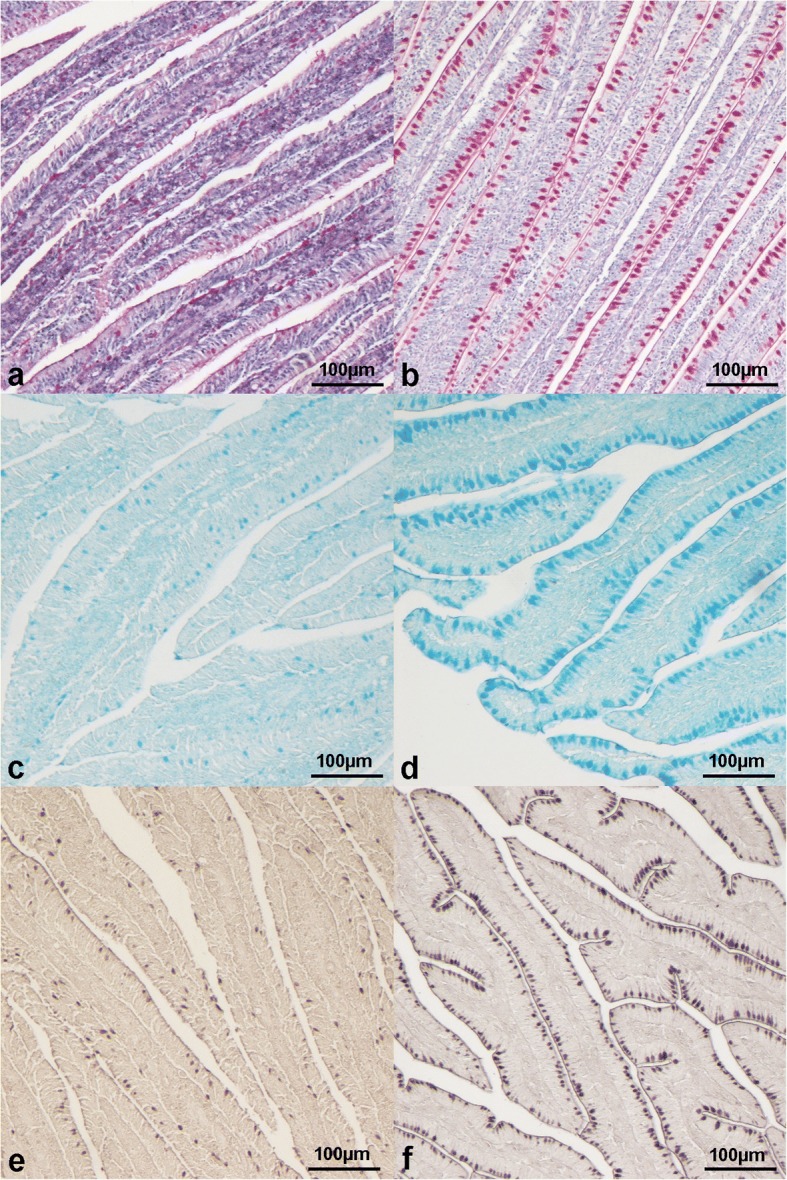
Histological pictures of duodenal (**a**, **c**, **e**) and ileal (**b**, **d**, **f**) villi stained with (**a**, **b**) periodic-acid Schiff (10× magnification), (**c**, **d**) Alcian Blue pH 2.5 (10× magnification) and (**e**, **f**) high iron diamine (10× magnification). Ileal villi show higher mucin staining intensity than duodenal ones

There was no significant effect of dietary TM meal inclusion or mucin type (*P* > 0.05) on mucin staining intensity for the intestinal villi, whereas both gut segment and villus fragment significantly influenced (*P* < 0.001 and *P* < 0.01, respectively) the histochemical findings (Table 1). In particular, villi showed higher mucin staining intensity in the ileum (*P* < 0.001) compared with the other gut segments and in the jejunum (*P* < 0.001) compared with the duodenum, respectively (Fig. 7). Lower mucin staining intensity was also observed in the villus tip (*P* = 0.001) than the base (Table 2).

## Discussion

A clear definition for “gut health” that encompasses all the intestinal physiological and functional features, such as nutrient digestion and absorption, host metabolism and energy generation, a stable microbiome, mucus layer development, barrier function, and mucosal immune responses, has never been reported [1]. However, the crosstalk between the diet composition, intestinal barrier (formed by the mucus layer together with the epithelial cell layer) and intestinal microbiota seems to be crucial for gut health determination [2]. For such reasons, several authors have recently focused their attention on the relationship between gut microbiota and mucin composition, gut microbiota and morphology, or gut morphology and mucin composition when dietary modifications occurred in poultry. However, systematic studies about the assessment of gut health by the evaluation of all the three aspects together (gut microbiota, morphology and mucin composition) in chickens facing to new diets are still very limited. Furthermore, no information about the modulation of intestinal microbiota and mucin composition by dietary insect meal inclusion in poultry are currently available.

### Cecal microbiota characterization

Characterization of the cecal microbiota of free-range poultry has already been reported in previous studies about Chinese Dagu chickens [22], Italian local breeds [23] and Bermuda free-range chickens [24]. However, the present study is the first one investigating the cecal microbiota of Label Hubbard chickens fed with different diets. The attention was herein focused on cecum as representative gut segment, since it harbors the highest microbial cell densities (up to 10^11^ cells/g) and diversity, has the longest residence time (12–20 h) of digesta in the gastrointestinal tract, and is an important site for recycling of urea, water regulation, and carbohydrate fermentations contributing to intestinal health and nutrition [25].

In the free-range chickens fed with basal diet of the present study, cecal microbiota was mainly colonized by the phyla *Bacteroidetes*, *Firmicutes* and *Proteobacteria*, as previously reported in normal chickens [25–28]. In particular, the predominance of the phylum *Bacteroidetes* over *Firmicutes* and *Proteobacteria* is in agreement with the previous findings about cecal microbiota of Dagu [22] and Bermuda [24] free-range chickens. A more similar prevalence of *Bacteroidetes*, *Firmicutes* and *Proteobacteria* phyla was observed in Italian local free-range breeds, but a comparative reduction of *Firmicutes* and a concomitant increase of *Bacteroidetes* was still identified in these birds when compared to broilers [23].

Differently from phyla, at genus level there is some controversy over the predominant taxa in chicken cecal microbiota. The majority of studies observed that the most predominant genera found in chicken cecum are *Clostridium*, *Ruminococcus*, *Lactobacillus* and *Bacteroides* [28–32], while Callaway et al. [33] also identified *Prevotella* as the most abundant genus. In particular, *Bacteroides* genus, U.m. of *Prevotellaceae* family, U. m. of *Ruminococcaceae* family and U. m. of *Bacteroidales* order have been reported as the most predominant members of cecal microbiota of Bermuda free-range chickens [24]. The current trial, which observed *Bacteroides*, U. m. of *Bacteroidales* order, *Clostridium* and *Ruminococcus* as the most predominant genera, can fit into the overall view of these previous studies.

With regards to gut microbiota modulation by insect meal utilization, the cecal microbiota of the free-range chickens of the present study showed higher α- and β-diversity when fed with TM meal compared to C feed. This represents a quite relevant finding, since high levels of diversity have been reported to help maintain the stability of intestinal microbiota after environmental stress [34] and determine the colonization resistance against invading pathogens [35]. Another interesting aspect to consider is that the average Shannon index identified in the current trial was about 8. This is in contrast with the previous studies about the chicken cecal microbiota, which reported a Shannon index varying from 3 to 6 [36, 37]. Mancabelli et al. [23] already pointed out that free-range chickens displayed a higher level of complexity of the gut microbiota compared to that found in broilers, with a Shannon index slightly higher than 6. Interestingly, Ferrario et al. [24] also observed similar microbial diversity between free-range and feral (i.e., formerly domesticated, wild-living) chickens, with the last being characterized by a Shannon index around 7. Therefore, it could be hypothesized that the semi-wild and wild rearing conditions may progressively modulate the complexity of the intestinal microbiota, thus underlying the role of environment and human influence on the bacterial communities within the chicken gastrointestinal tract.

Similarly to what observed for C birds, the cecal microbiota of the free-range chickens fed with TM diet of the present study was mainly colonized by the phyla *Bacteroidetes*, *Firmicutes* and *Proteobacteria*. Furthermore, the free-range chickens fed with TM showed *Bacteroides*, U. m. of *Bacteroidales* order, *Clostridium* and *Ruminococcus* as the most predominant genera of their cecal microbiota. These findings suggest that TM meal utilization does not alter the physiological cecal community at both phylum and genus levels.

Investigating the compositional differences in the cecal microbiota between C and TM birds, the free-range chickens fed with insect meal showed increased and decreased abundances of *Firmicutes* and *Bacteroidetes* phyla, respectively, along with higher *Firmicutes:Bacteroidetes* ratios. This represents a quite relevant finding, since bacteria within *Firmicutes* phylum have an important role in the digestion of feed and the host health [38] and greater *Firmicutes:Bacteroidetes* ratios have been associated with bacterial profile with higher capacity of energy harvesting [39]. At genus level, the free-range chickens fed with TM showed an increase in the abundance of *Clostridium*, *Oscillospira*, *Ruminococcus, Coprococcus* and *Sutterella* genera in their cecal community. As already pointed out, *Clostridium* and *Ruminococcus* are some of the most predominant genera found in chicken cecum [28–30]. On the other hand, *Clostridium* genus, along with *Oscillospira* and *Coprococcus*, also encompasses bacteria capable of producing butyrate [40, 41]. Butyrate has been demonstrated to have a positive role on growth performance, intestinal villus structure and pathogen control, as well as anti-inflammatory properties [42]. Furthermore, bacteria belonging to *Ruminococcus* genus can also produce other short chain fatty acids (i.e., acetic and succinic acid) through glucose metabolism and digest cellulose in food [43]. It is well known that short chain fatty acids are an important source of energy for enterocytes and are vital for intestinal health [44]. Another interesting finding of the cecal community of the birds fed with TM in the current trial is the identification of increased abundance of *Sutterella* genus*,* which has been reported to be a potential probiotic present in pigeon “milk” that can improve the rate of growth and feed conversion ratio in chickens [45]. The increase of the above mentioned bacterial taxa suggests that TM meal utilization may positively modulate the cecal microbiota of birds. In particular, the increase of butyrate- and short chain fatty acids-producing bacteria may have important implications. However, further studies also evaluating the microbial metabolites and metabolic pathways are needed to better contextualize these OTUs changes.

Despite this overall positive modulation, the reduction of *Bacteroides* genus within the cecal community of the free-range chickens fed with TM meal of the present study may be considered a potential negative finding. Indeed, apart from being one of the most predominant genera found in chicken cecum [28, 31, 33], this taxon has been reported to be an important contributor to the intestinal health of the birds, because of its beneficial role for weight gain and growth performance [46] and the inhibition of *Clostridium perfringens* sporulation by its fermentation products [47]. As a consequence, depletion of *Bacteroides* genus could be problematic for chickens, since it may predispose the animal gut to *Clostridium perfringens* infection and gastroenteritis [47]. However, *Bacteroides* genus still outnumbered the other taxa in TM group of the present study. The birds fed with insects also showed similar growth performance to those fed with C diet [21], thus further mitigating this potential negative result. As a final aspect to underline, in spite of the overall positive cecal microbiota modulation due to TM meal utilization, the growth performance of the chickens remained unaffected. However, since a clear cause-effect relationship between diversity and composition of cecal microbiota and bird performance has not yet been confirmed, the gut microbiota findings need to be contextualized with those related to mucin composition and morphology.

### Intestinal morphology

Dietary TM meal inclusion in free-range chickens of the current trial did not affect the gut morphometric indices, as already reported in details by Biasato et al. [21]. Features of the gastrointestinal tract have been reported to influence the efficiency of utilization of dietary protein [48], which is considered a crucial regulator of poultry growth and reproductive performance [7]. In particular, the microscopic structure of small intestine in terms of villus height and crypt depth is considered the main indicator of intestinal development, health and functionality, such influencing nutrient digestion and absorption [5]. Particular attention is given to crypt and villus since the former is the region in which new intestinal cells are formed and the latter has a fundamental role in the digestion and absorption of nutrients [48]. The ideal gut morphological asset appears to be characterized by long villi and shallow crypts. Indeed, longer villi are generally associated with increased total luminal absorptive area and subsequent satisfactory digestive enzyme action and higher transport of nutrients [7]. In parallel, shallower crypts reflect the prolonged survival of villi without the need for renewal [49], with reduced energy expenditure for this process and consequent enabled growth of other tissues [50]. Since no standardized measurements ranges referring to “long villi” and “shallow crypts” have been determined till now, the identification of unaffected gut morphometric indices and growth performance in the birds fed with insects of the present study is enough to suggest that TM meal does not alter the physiological intestinal morphology and subsequently the digestion efficiency.

Independently of TM meal utilization, the duodenum of the free-range chickens of the current trial showed greater development in relation to the other gut segments. It is well known that the intestine possesses an inherent ability to create and maintain regional differences with regard to mucosal structure [51], thus determining different absorption processes depending on the segment considered [52]. The identification of a proximodistal decreasing gradient of the morphometric indices from the duodenum to the ileum, which is in agreement with the previous studies [4, 52, 53], is another important aspect in terms of preservation of the physiological gut development and absorption processes.

### Intestinal mucin composition

The present study is the first one to investigate the effects of dietary TM meal inclusion on gut mucin composition in poultry. Furthermore, the characterization of the three types of mucins (i.e., neutral, acidic sialylated and acidic sulfated) in the two intestinal mucosal compartments (i.e., crypts and villi) of four defined segments (i.e., duodenum, jejunum, ileum and cecum) represents a unique histochemical approach.

Dietary TM meal inclusion in free-range chickens of the current trial did not influence the mucin staining intensity either in the intestinal crypts or villi. This finding could reflect an innate feeding habit, since chickens naturally consume insects when reared in free-range systems [54].

Independently of TM meal utilization, the intestinal crypts of the birds of the current trial showed greater acidic sialylated mucins staining intensity. This represents an interesting finding, since sialic acid groups have protective properties [55] and increase in acidic sialylated mucins production has been hypothesized to represent a defense strategy against mucus degradation by bacterial colonization [4]. On the contrary, similar staining intensity of the three types of mucins was observed in the intestinal villi. The variations in the proportion of the goblet cells containing the mucin types observed in the intestinal crypts instead of villi could be related to the higher presence of mucin-producing goblet cells in the first compartment, as reported by Uni et al. [56].

The intestinal crypts of both chickens fed with control and TM diets of the present research showed lower mucin staining intensity in the cecum compared with the other gut segments. This finding is in agreement with what observed by Tsirtsikos et al. [9], even if the authors provided no explanation for that result. The avian cecum has been found to be a site for fermentation and further digestion of feed (especially for breakdown of cellulose), for utilization and absorption of water and nitrogenous components, for microbial action of both beneficial and disease-causing organism, and as a site for production of immunoglobulins and antibodies [57]. As previously reported mucins enhance the propulsion of chyme, modulate nutrient absorption and protect the intestinal mucosa from enteric pathogens [6]. Since the small intestine represents the major site of nutrient absorption [58] and the ileum has been suggested to be more predisposed to bacterial colonization [4], it is possible to speculate that the mucin synthesis for nutrient metabolism and gut protection could be reduced in the cecum. Furthermore, because the cecum is blind-ended, its contents can be retained for long periods, with no need for mucin secretion to facilitate the propulsion of chyme. Therefore, the decreased mucin staining intensity in the cecal crypts observed in the present study could be considered a physiological feature related to the different anatomy and physiology of the cecum.

Independently of dietary TM meal inclusion, the intestinal villi of the free-range chickens of the present study showed greater mucin staining intensity in the ileum compared with the other gut segments. This is consistent with previous findings in chickens [4, 59], which demonstrated a distal increase in the density of goblet cells along the duodenal-ileal axis [59]. Forder et al. [4] suggested that the distal ileum could be a preferred region for bacterial colonization, thus explaining the need for greater protection and subsequent higher mucin synthesis.

The intestinal crypts of both birds fed with control and TM diets of the current trial showed greater mucin staining intensity in the base compared with the other crypt fragments. This is in agreement with previous studies, in which the decreased stain in the crypt tip has been suggested to be related to the process of proliferation and maturation of goblet cells [9, 56]. Similarly, the intestinal villi showed higher mucin staining intensity in the base compared with the other villus fragments. This is in contrast with what observed by Tsirtsikos et al. [9, 10], which found higher staining intensity at the villus tip and explained this increased accumulation as a confirm for the key role of mucin in the protection of gut epithelium against luminal threats [9, 10]. However, the process of cell proliferation in chicken intestinal epithelium has also been reported to occur along the entire length of the villus, with proliferation activity decreasing from crypt to the upper half of the villus [56]. Therefore, the higher mucin staining intensity observed at the villus base in the birds of the present study can be related to the physiological goblet cells proliferation process occurring in villus compartment.

## Conclusions

In conclusion, dietary TM meal inclusion may positively modulate the gut microbiota of the free-range chickens without influencing the intestinal morphology and mucin composition. The identification of physiological cecal community, gut morphological development and mucin dynamics also suggests that insect meal utilization does not negatively affect the gut health of the birds. Furthermore, since the rapid growth of chickens directly depends on morphological and functional integrity of the digestive tract (as herein confirmed), the gut health assessment by a post mortem multidisciplinary approach appears to be fundamental.

## Methods

### Birds and experimental design

The present study is a part of ongoing research work that aims to investigate the effects of TM meal inclusion on growth performance, haematochemical profile, carcass traits, histological features and gut health of free-range chickens [21]. In order to avoid unnecessary repetition of already published data, a brief summary of the experimental trial is reported below. The experimental protocol was approved by the Ethical Committee of the Department of Veterinary Sciences of the University of Turin [Italy].

A total of 140 43-days-old female Label Hubbard hybrid chickens (female: JA 57 × male: S77CN, mean weight: 716.3 ± 23.2 g, purchased from “Aglietto Natura SRL” farm [Bianzè, VC – Italy]), a medium-growing genotype, were randomly allotted to two dietary treatments. Each of them consisted of five replicate floor pens, with 14 chicks per pen. The experimental unit was the pen. Two diets were formulated: a control corn-soybean-gluten meal-based diet (C), normally used by the breeder, and an experimental diet (TM7.5), in which full-fat TM larvae meal (Gaobeidian Shannong Biology Co. Ltd., Gaobeidian, Hebei province - China) was included at 75 g/kg in complete substitution of corn gluten meal. Details of the diets are shown in Additional file 3. Nutrients digestibility, apparent metabolisable energy and nitrogen-corrected apparent metabolisable energy were previously assessed [17]. All the birds were free-range reared under the same environmental conditions throughout the experimental trial. Feed (isonitrogenous and isoenergetic diets) and water were provided ad libitum for the whole experimental periods. The chickens (showing overall good health conditions at the beginning of the experiment) were regularly vaccinated and showed no signs of illness or mortality throughout the trial. Growth (initial and final live weight, average feed intake and feed conversion ratio) and slaughtering performance (carcass, breasts, thighs, deboned thighs, spleen, bursa of Fabricius, liver, gizzard and abdominal fat weights) were also unaffected by dietary treatments. Welfare-related assessments (footpad dermatitis score evaluation) were also performed after the experimental trial. The experiment lasted 54 days.

### Intestinal sampling and processing

A total of ten chickens per treatment (two birds per pen) were randomly selected and slaughtered in a commercial abattoir at 97 days of age. Animals were euthanized by electrical stunning and bleeding. The remaining birds were slaughtered using the same euthanasia procedures and carcasses were submitted to proper disposal methods. Cecal content was collected into sterile plastic tubes that were promptly refrigerated (for a maximum of 2 h) and frozen at − 80 °C until DNA extraction. Intestinal segment samples (approximately 5 cm in length) of duodenum, jejunum, ileum and cecum were excised and flushed with 0.9% saline to remove all the content. The collected segments of intestine were the loop of the duodenum (duodenum), the tract before Meckel’s diverticulum (jejunum), the tract before the ileocolic junction (ileum) and the apex of the caeca (cecum). Gut segments were fixed in Carnoy’s and 10% buffered formalin solutions for morphometric analysis and histochemical staining, respectively. Tissues were routinely embedded in paraffin wax blocks, sectioned at 5 μm thickness and mounted on glass slides.

### DNA extraction and sequencing

A pool of the cecal content from two chickens per pen (five pools per feeding group) was submitted to DNA extraction and sequencing. DNA was extracted with a commercial kit (DNAzol® Reagent, Thermo Fisher Scientific) according to the manufacturer’s instructions. One μl of RNase (Illumina Inc., San Diego, CA) was added to digest RNA in the DNA samples, with an incubation of 1 h at 37 °C. DNA was quantified using the NanoDrop and standardized at 5 ng/μl. Microbial diversity was studied by sequencing the amplified V3-V4 region of the 16S rRNA gene by using primers and PCR conditions previously reported [60]. Samples multiplexing, library purification pooling and sequencing was carried out as described in the “16S Metagenomic Sequencing Library Preparation” guide by Illumina. Libraries were sequenced by BMR genomics (Padova, Italy) on a MiSeq platform (Illumina Italy s.r.l., Milan, Italy), leading to 250 bp, paired-end reads. After the first purification step following the Illumina sample preparation procedure, the library was combined with the sequencing adapters and dual indices using the Nextera XT Index Kit (Illumina, San Diego, USA), obtaining the multiplexed paired-end libraries. Individual libraries concentration in nM were calculated based on the size of amplicons by using a Bioanalyzer (Agilent) and diluted to 4 nM, denaturated with 0.2 N NaOH and spiked with 20% (*v*/v) of PhiX.

### Histomorphological investigations

Carnoy-fixed and paraffin-embedded intestinal sections of ten chickens per feeding group (two birds per pen) were submitted to Haematoxylin & Eosin staining and one slide per each intestinal segment was examined by light microscopy. Each slide was captured with a Nikon DS-Fi1 digital camera coupled to a Zeiss Axiophot microscope using a 2.5× objective lens. NIS-Elements F software was used for image capturing. Morphometric analysis was performed by Image®-Pro Plus software. The evaluated morphometric indices were villus height (Vh, from the tip of the villus to the crypt), crypt depth (Cd, from the base of the villus to the submucosa) and the villus height to crypt depth ratio (Vh/Cd) [7]. Morphometric measurements were performed on 10 well-oriented and intact villi and 10 crypts chosen from duodenum, jejunum and ileum [8].

### Histochemical staining

Formalin-fixed and paraffin-embedded intestinal sections of ten chickens per feeding group (two birds per pen) were submitted to three different histochemical staining, in order to characterize the three types of mucins [4].

Neutral mucins were identified by periodic-acid Schiff staining. Sections were brought to water, immersed in 0.5% periodic acid solution for 20 min, washed in running tap water for 5 min and immersed in Schiff’s reagent for a further 30 min. Sections were successively rinsed in running tap water for 10 min, dehydrated and mounted. Neutral mucins stained magenta [61].

Acidic sialylated mucins were identified by Alcian Blue pH 2.5 staining. Sections were brought to water, immersed in 8 G X alcian blue in 3% acetic acid solution for 30 min, washed in running tap water for further 5 min and successively dehydrated and mounted. Acidic sialylated mucins stained blue [62].

Acidic sulfated mucins were identified by high iron diamine staining. Sections were brought to water, oxidized in 1% periodic acid solution for 10 min and washed in running tap water for further 5 min. Sections were successively immersed in the high iron diamine solution (120 mg metadiamine, 20 mg paradiamine and 1.4 ml 10% ferric chloride in 50 ml distilled water) for 18 h, rinsed very rapidly in tap water, dehydrated and mounted. Acidic sulfated mucins stained purple-black [63].

### Mucin staining intensity

One slide per histochemical staining for each intestinal segment was examined by light microscopy. A total of 10 crypts and 10 villi for each slide were evaluated. Crypts and villi were divided into three fragments (base, midsection and tip), according to Tsirtsikos et al. [9, 10]. Mucin staining intensity of goblet cells was scored semiquantitatively for each fragment as follows: grade 0 for absent staining, grade 1 for mild staining, grade 2 for moderate staining and grade 3 for marked staining. The score was formulated according to that proposed by Tsirtsikos et al. [9, 10] for the mucus layer and depended on the number of positive goblet cells and the intensity of the staining. All the slides were assessed blinded by three observers (IB, EB and MTC) and the discordant cases were reviewed at a multi-head microscope until a consensus was reached.

### Bioinformatics and statistical analysis

The experimental unit was the pen for 16S rRNA sequences and bird for morphometric and histochemical data.

Paired-end reads were first merged using FLASH software [64] with default parameters. Joint reads were further quality filtered (at Phred > Q20) using QIIME 1.9.0 software [65] and the pipeline recently described [66]. Briefly, operational taxonomic units (OTUs) were picked at 97% of similarity by means of UCLUST clustering methods [67] and representative sequences of each cluster were used to assign taxonomy using the Greengenes 16S rRNA gene database (version 2013). Alpha diversity indices were calculated using the *diversity* function of the vegan package [68]. A pairwise t-test was used to assess differences between the theses. The diversity indices were further analyzed by pairwise comparisons using Wilcoxon rank sum test to assess differences between the dietary treatments. Weighted UniFrac distance matrices were used to perform Adonis and ANOSIM statistical tests in R environment. A filtered OTU table was generated at 0.1% abundance in at least 2 samples through QIIME. The table was then used to produce the Principal Component Analysis (PCA) in R environment (https://www.r-project.org). OTU table showed the higher taxonomy resolution reached by the 16S data. When the taxonomy assignment was not able to reach the genus level, the phylum, class, order or family were displayed. Pairwise Kruskal-Wallis tests were used to find significant differences in microbial taxa abundance according to the dietary treatment. The experimental unit was the pen. *P*-values were adjusted for multiple testing and a false discovery rate (FDR) < 0.05 considered as significant.

The statistical analysis of morphometric and histochemical data was performed using IBM SPSS Statistics V20.0.0 software. The influence of diet on intestinal morphometric measurements was tested using Student’s *t* test for independent samples. Morphometric data were also analyzed by means of One-way ANOVA (post hoc test: Duncan’s multiple range test) to evaluate the influence of intestinal segment within each dietary treatment. Results were expressed as mean and pooled standard error of the mean (SEM). *P* values < 0.05 were considered statistically significant [21]. Histochemical data were analyzed by fitting a generalized linear model (GLM) similar to those proposed by Tsirtsikos et al. [9, 10]. The GLM allowed the mean mucin staining intensity scores to depend on linear predictors such as diet, mucin type, intestinal segment and fragment within crypts or villi through a negative binomial response probability distribution with a nonlinear link function (log). The bird within treatment effect was also included in the GLM as the repeated factor. A hybrid method for parameter estimation was used and a type III analysis with Wald chi-square test was applied to assess the model effects. Results were expressed as least squares means and SEM and the interactions between factor levels were evaluated by pairwise comparisons. P values < 0.05 were considered statistically significant. Statistical analysis was performed by procedure “Generalized Linear Models”.

## Additional files

Additional file 1: Good’s coverage and α-diversity measures of cecal microbiota of free-range chickens fed with control (C) and 7.5% inclusion level of *Tenebrio molitor* meal (TM7.5) diets. Description column indicates the 5 replicate pens of control (C_1, C_2, C_3, C_4 and C_5) and 7.5% inclusion level of *Tenebrio molitor* meal (TM7.5_1, TM7.5_2, TM7.5_3, TM7.5_4 and TM7.5_5) dietary treatments. (XLSX 9 kb)

Additional file 2: Relative abundance of the main bacterial phyla and genera of cecal microbiota of free-range chickens fed with control (C) and 7.5% inclusion level of *Tenebrio molitor* meal (TM7.5) diets. (XLSX 10 kb)

Additional file 3: Ingredients and chemical composition of the experimental diets. The mineral-vitamin premix (Trevit Volatili 3.5 - Trei - Rio Saliceto (RE) Italy) given values are supplied per kg: 650.000 IU of vitamin A; 65.000 IU of vitamin D3; 650 IU of vitamin E; 80 mg of vitamin K; 80 mg of vitamin B1; 150 mg of vitamin B2; 770 mg of vitamin B3; 80 mg of vitamin B6; 0.5 mg of vitamin B12; 240 mg of pantothenic acid; 4700 mg of betaine; 1750 mg of Iron (II) carbonate; 1835 mg of Magnesium oxide; 1612 mg of Zinc oxide; 178 mg of Copper (II) oxide; 18.3 mg of Potassium iodide; 6.6 mg of Sodium selenite; 4100 mg of DL-methionine; 5500 mg of L-lysine; 120 g Calcium carbonate; 450 g Calcium phosphate; 11.5 g of Sodium chloride. SFA: saturated fatty acids; MUFA: monounsaturated fatty acids; PUFA: polyunsaturated fatty acids. Other fatty acids (all less than 0.40 g/kg DM): C12:0, C14:0, C14:1 *cis*9, C16:1 *cis*9, C18:1 *cis*11, C20:0, C18:3 n6, C20:1 *cis*9, C20:1 *cis*11. TM, *Tenebrio molitor*; AME, apparent metabolizable energy; DM, dry matter; CP, crude protein; C = control; TM7.5 = 7.5% inclusion level of *Tenebrio molitor. (XLSX 10 kb)*

## Abbreviations

C: Control
Cd: Crypt depth
FDR: False discovery rate
GLM: Generalized linear model
OTUs: Operational taxonomic units
PD: Phylogenetic Diversity
TM: *Tenebrio molitor*
TM7.5: 7.5% inclusion level of *Tenebrio molitor*
Vh: Villus height
Vh/Cd: Villus height to crypt depth ratio
